# Cobalt Single-Atom/Nanoparticle Dual Sites on Unzipped Carbon Nanotubes for Electrolyte-Switchable Glucose and Uric Acid Sensing

**DOI:** 10.3390/s26216777

**Published:** 2026-10-26

**Authors:** Alfi Rodiansyah, Changhyun Do, Sae Yane Paek, Jong Min Kim, Joonwon Lim

**Affiliations:** 1 Department of Information Display, College of Sciences, Kyung Hee University, Seoul 02447, Republic of Korea; rodiansyahalfi@khu.ac.kr; 2 Laboratoire de Physique de la Matière Condensée, CNRS, École Polytechnique, Institut Polytechnique de Paris, 91128 Palaiseau, France; 3 Extreme Materials Research Center, Korea Institute of Science and Technology, Seoul 02792, Republic of Koreajongminkim@kist.re.kr (J.M.K.); 4 KHU-KIST Department of Converging Science and Technology, Kyung Hee University, Seoul 02447, Republic of Korea

**Keywords:** single atom/nanoparticle, electrolyte-regulated sensor, carbon complex, unzipped CNT, glucose sensing, UA sensing, multi-analyte detection

## Abstract

Non-enzymatic multi-analyte sensing using a single electrode remains challenging owing to the distinct electrochemical environments and catalytic pathways required for different biomarkers. Here, we report an electrolyte-regulated bifunctional electrocatalyst comprising atomically dispersed Co-N/O sites and Co/CoO nanoparticles anchored on nitrogen-doped unzipped carbon nanotubes (SA/NP Co@N-UzCNT) for selective glucose and uric acid (UA) detection. Partial unzipping of the outer CNT walls generates edge-rich graphene nanoribbons while preserving the electrically conductive inner CNT framework, providing abundant N-coordination sites for stabilizing structurally distinct Co motifs. Under alkaline conditions, nanoparticle-derived Co sites are electrochemically converted into catalytically active CoOOH species that promote glucose oxidation, whereas atomically dispersed Co-N sites dominate UA oxidation under neutral conditions, demonstrating electrolyte-dependent catalytic selectivity between the two Co configurations. Switching the electrolyte environment alters the dominant catalytic site, enabling selective detection of glucose and UA using a single-electrode architecture. The resulting sensor achieves sensitivities of 65.18 µA mM^−1^ cm^−2^ for glucose in the concentration range of 1–8 mM and 337.5 µA mM^−1^ cm^−2^ within the concentration range of 5–500 µM for UA. Practical applicability is demonstrated using a screen-printed electrode strip, which retains approximately 89% of its initial glucose response after 15 days. This work demonstrates that single-atom/nanoparticle coexistence can serve as a functional catalytic architecture for electrolyte-regulated non-enzymatic multi-analyte sensing.

## 1. Introduction

Glucose and uric acid (UA) are important biomarkers of metabolic homeostasis, providing complementary information on carbohydrate and purine metabolism [1,2,3,4,5]. Under normal physiological conditions, fasting blood glucose is generally maintained below 100 mg dL^−1^ (approximately 5.6 mM), whereas fasting levels of 100–125 mg dL^−1^ (5.6–6.9 mM) are associated with prediabetes and values of ≥126 mg dL^−1^ (≥7.0 mM) are within the diagnostic range for diabetes mellitus. Persistent dysregulation of blood glucose is therefore a defining feature of impaired glucose metabolism and diabetes. In contrast, as an important metabolic biomarker, the normal concentration of uric acid (UA) in human blood ranges from 15 to 80 mg L^−1^ (approximately 100 µM to 500 µM). In contrast, UA concentrations as low as 6 mg L^−1^ (approximately 35.7 µM) have been reported in patients with urate nephropathy and gout. Abnormal UA levels are associated not only with gout, chronic kidney disease and metabolic syndrome, but also with diabetes-related complications [6,7,8,9,10,11,12]. Because disturbances in glucose and UA metabolism frequently coexist in metabolic disorders, their simultaneous monitoring could provide a more comprehensive assessment of metabolic status than either measurement alone. A sensing platform capable of detecting both analytes using a single, low-cost electrode would therefore be highly desirable for decentralized and point-of-care diagnostics [13,14,15,16].

Commercial glucose and UA sensors predominantly rely on enzymes such as glucose oxidase and uricase, which provide high molecular selectivity through specific biorecognition [17,18]. However, their performance critically depends on preservation of the native enzyme structure and is consequently susceptible to temperature, pH, humidity, and storage conditions [19,20,21,22]. These challenges are particularly pronounced in multi-analyte sensing, where co-immobilization of multiple enzymes within a common electrode architecture requires simultaneous preservation of their stability and compatible reaction environments. Non-enzymatic electrochemical sensing offers an attractive alternative by directly coupling analyte oxidation to catalytically active surfaces [23,24]. Despite these advantages, extending non-enzymatic electrochemical sensing to multiple analytes remains challenging owing to the fundamentally different electrochemical environments required for glucose and UA oxidation. On many transition-metal-based catalysts, glucose oxidation is promoted under strongly alkaline conditions through surface oxyhydroxide-mediated redox processes. In contrast, UA can be efficiently oxidized at neutral pH through direct electron-transfer pathways, where excessive surface oxidation or hydroxide-derived intermediates can hinder selective detection. This mismatch in the preferred reaction environments makes single-electrode, non-enzymatic detection of glucose and UA intrinsically challenging [25,26,27,28,29,30,31].

Single-atom catalysts (SACs) provide an intriguing opportunity to address this challenge through atomic-level regulation of the local coordination environment [32,33]. However, the intrinsically high surface energy of isolated metal atoms drives their migration and aggregation, making nanoparticle formation a prevalent route toward the loss of atomic dispersion. This conventional interpretation overlooks the potential advantages of mixed atomic configurations [34]. Single atoms (SAs) and nanoparticles (NPs) composed of an identical metal species can exhibit markedly different electronic structures, adsorption characteristics, and redox behavior, enabling chemically different catalytic pathways [35,36]. The deliberate integration of atomically dispersed and nanoscale metal sites within a common conductive scaffold offers a route to complementary catalytic functions selectively activated by the reaction environment. Although SA/NP combinations have attracted increasing attention in electrocatalysis, most studies have focused on energy-conversion reactions or the enhancement of a single catalytic process [37,38]. The use of SA/NP dual-site catalysts for electrochemical multi-analyte sensing remains largely unexplored.

Here, we develop an electrolyte-switchable bifunctional electrocatalyst comprising cobalt single atoms and nanoparticles anchored at the edges of nitrogen-doped unzipped carbon nanotubes (SA/NP Co@N-UzCNT) for non-enzymatic glucose and uric acid sensing, where the Co single-atom sites primarily facilitate the uric acid oxidation reaction under neutral conditions, while the Co-containing nanoparticles promote glucose oxidation under alkaline conditions. Selective partial unzipping of the outer CNT walls generates edge-rich graphene nanoribbons while retaining the conductive inner CNT framework. Nitrogen functionalities concentrated along the nanoribbon edges provide abundant coordination sites that stabilize atomically dispersed Co centers while concurrently accommodating nanoscale Co/CoO particles. These structurally distinct Co motifs exhibit electrolyte-dependent catalytic selectivity through the transformation of Co NPs into catalytically active CoOOH species under alkaline conditions for glucose oxidation, whereas atomically dispersed Co-N/O sites dominate UA oxidation under neutral conditions. By integrating these complementary catalytic motifs within a single conductive architecture, the dominant active site can be switched through the electrolyte environment, enabling selective detection of glucose and UA on a common electrode. This sensing strategy is implemented in a dual-analyte strip-type sensor, demonstrating electrolyte-regulated non-enzymatic multi-analyte sensing.

## 2. Materials and Methods

### 2.1. Materials

Multiwalled carbon nanotubes (MWCNTs, diameter ~17 nm) purchased from Hanwa Nanotech, potassium permanganate, concentrated sulfuric acid, hydrogen peroxide, hydrazine monohydrate, cobalt (II) chloride hexahydrate, sodium hydroxide, D-(+)-glucose, uric acid, ascorbic acid, urea, sodium chloride and potassium chloride, and phosphate-buffered saline were purchased from Sigma-Aldrich (St. Louis, MO, USA) and used as received. All aqueous solutions were prepared with deionized water (18.2 MΩ cm).

### 2.2. Partial Unzipping of MWCNTs (UzCNT)

MWCNTs were partially unzipped by a modified oxidative longitudinal cutting route. An amount of 1 g of MWCNTs was dispersed in 240 mL of concentrated H_2_SO_4_ and stirred for 1 h at room temperature, after which 5 g of KMnO_4_ was added in portions, and the suspension was heated at 65 °C for 1.5 h. The reaction was quenched with ice water containing H_2_O_2_, and the product was washed to neutral pH by repeated filtering and freeze-dried.

### 2.3. Synthesis of N-Doped Unzipped CNT (N-UzCNT)

An amount of 400 mg of UzCNT was dispersed in 100 mL of deionized water; to it, 120 mL of hydrazine monohydrate was added, and the mixture was refluxed at 95 °C for 24 h. The solid was collected, washed with water/ethanol and freeze-dried.

### 2.4. Synthesis of SA/NP Co@N-UzCNT

An amount of 100 mg of N-UzCNT was dispersed in 50 mL of DI water containing 5 mg of CoCl_2_·6H_2_O. The solution was stirred for 1 h, followed by a hydrothermal process at 180 °C for 24 h to allow coordination of Co ions to the nitrogen-rich edges. After solvent removal, the powder was pyrolyzed at 700 °C for 1 h under flowing NH_3_ 60 sccm, then cooled to room temperature under Ar.

### 2.5. Characterization

Morphology was examined by field-emission SEM Hitachi S-4800 (Hitachi, Tokyo, Japan); aberration-corrected HAADF-STEM imaging and EDS elemental mapping were performed on a Titan 800 (Thermo Fisher Scientific, Eindhoven, The Netherlands) operated at 300 kV. XRD patterns were collected on an X’Pert PRO/Malvern PANalytical (Malvern Panalytical, Almelo, The Netherlands) with Cu Kα radiation. Raman spectra were recorded with a 532 nm excitation laser in Via Reflex/Renishaw (Renishaw plc, Wotton-under-Edge, UK). XPS was carried out on a PHI 5000 Versa Probe (ULVAC-PHI, Inc. Chigasaki, Japan) with monochromated Al Kα radiation, and binding energies were calibrated to the C 1 s peak at 284.6 eV. Co K-edge XANES and EXAFS spectra were collected at 1D XRS beamline of KIST-PAL, whose machine is located in the Pohang Accelerator Laboratory, in fluorescence mode; data were processed and fitted in the IFEFFIT package over the range of k = 3–10.5 Å^−1^ and R = 1–3 Å.

### 2.6. Electrochemical Measurements

All electrochemical measurements were performed on a BioLogic (BioLogic, Seyssinet-Pariset, France) potentiostat in a three-electrode cell with a 5.5 mm glassy carbon working electrode (2.3746 cm^2^), a Pt wire counter electrode and an Ag/AgCl (saturated KCl) reference electrode. Catalyst inks were prepared by dispersing 2 mg of catalyst in 200 mL of ethanol, 200 mL of DI water, and 20 mL of Nafion 5%. The solution was sonicated for 30 min to ensure the uniformity of the ink. An amount of 7.48 mL of ink was drop-cast onto the polished working electrode to give a loading of 150 mg cm^−2^. Glucose oxidation was evaluated in 0.1 M NaOH and UA oxidation in 0.01 M PBS (pH neutral, 7.4). Cyclic voltammograms were recorded at 50 mV s^−1^ unless stated otherwise; amperometric measurements were carried out at 0.40 V vs. Ag/AgCl under continuous stirring; DPV was performed with a pulse amplitude of 50 mV, pulse width of 50 ms and step potential of 5 mV. Electrochemical impedance spectra were acquired at 0.40 V vs. Ag/AgCl over 0.01–100,000 Hz with an amplitude of 5 mV. Double-layer capacitance was extracted from CVs recorded in the non-faradaic region at scan rates of 10–100 mV s^−1^, and the ECSA was obtained using a specific capacitance of smooth surface glassy carbon of 40 µF cm^−2^.

### 2.7. Fabrication of the Strip-Type Sensor

Commercial screen-printed electrodes (TE100AgCl, Zensor R&D Co., Ltd., Taichung, Taiwan) comprising a carbon working electrode (3 mm), an Ag/AgCl pseudo-reference and a Pt counter electrode were modified by drop-casting 5 mL of the catalyst ink onto the working electrode and drying at room temperature for 24 h. For dual-analyte operation, the same strip was rinsed with deionized water and dried between the neutral and alkaline measurements. The reproducibility test was conducted with the same electrode that was first used for glucose detection in KOH electrolyte. Afterward, the electrode was thoroughly rinsed with DI water, dried, and subsequently transferred to PBS for UA detection. The alternating sequence between glucose sensing in KOH and UA sensing was repeated for a total of 10 switching cycles.

## 3. Results

### 3.1. Synthesis of Single-Atom/Nanoparticle Cobalt on Carbon Complex UzCNTs

**Figure 1:**
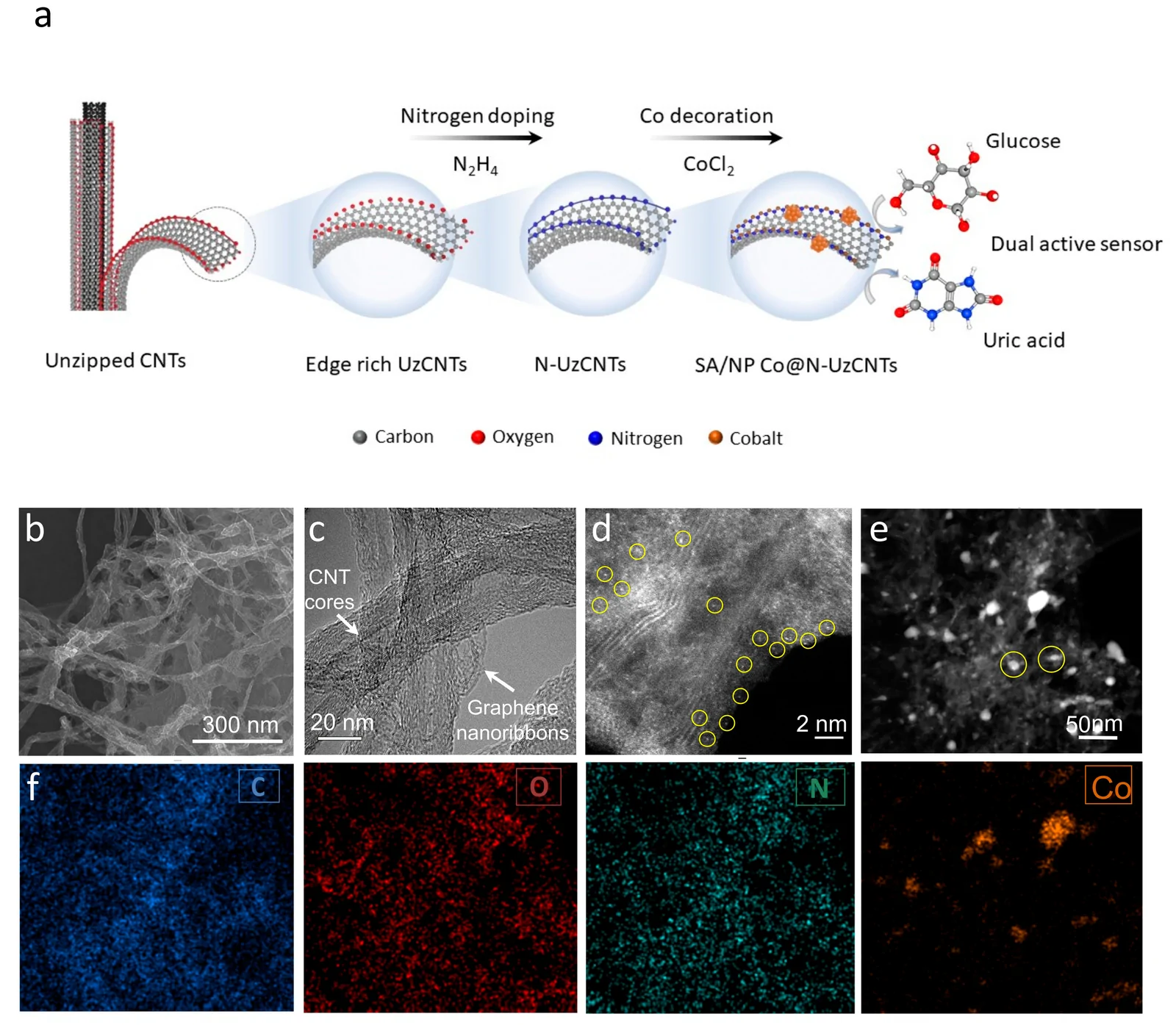
Morphology and structural characterization: (**a**) Schematic illustration of the synthetic process of SA/NP cobalt on nitrogen-doped unzipped CNT. (**b**) SEM image of unzipped CNT. (**c**) TEM image of unzipped CNT. (**d**) High-magnification aberration-corrected HAADF-STEM image of SA cobalt (yellow circle corresponds to single-atom cobalt). (**e**) High-magnification aberration-corrected HAADF-STEM image of SA/NP Co (yellow circle corresponds to particle cobalt). (**f**) The corresponding EDS elemental mapping of SA/NP Co@N-UzCNT.

Figure 1a schematically illustrates the synthesis of the SA/NP Co@N-UzCNT electrocatalyst through controlled partial unzipping of multiwalled carbon nanotubes (MWCNTs). The outer graphitic walls of the MWCNTs were selectively unzipped into graphene nanoribbons while the inner CNT cores remained largely intact, generating abundant edge sites without disrupting the continuous conductive framework. The newly formed ketone-rich nanoribbon edges were subsequently functionalized with hydrazine to introduce nitrogen functionalities preferentially along the exposed edges [39]. These N-rich sites served as coordination centers for Co precursors, promoting their immobilization within the unzipped carbon framework. Subsequent thermal treatment under an ammonia (NH_3_) atmosphere produced SA/NP Co@N-UzCNT containing both atomically dispersed Co sites and nanoscale Co particles. This hierarchical architecture combines edge-rich graphene nanoribbons, abundant N-coordination sites, and conductive CNT cores, providing a structurally integrated platform for stabilizing distinct Co active sites while maintaining efficient charge transport.

### 3.2. Characterization of Single-Atom/Nanoparticle Cobalt on UzCNTs

The morphology and microstructure of SA/NP Co@N-UzCNT were examined by scanning electron microscopy (SEM), transmission electron microscopy (TEM), and high-angle annular dark-field scanning transmission electron microscopy (HAADF-STEM). As shown in Figure 1b, the partially unzipped CNTs form an interconnected, porous network that preserves continuous conductive pathways while providing abundant edge sites as well as enlarged electrochemically accessible surfaces. TEM images (Figure 1c and Figure S2) reveal graphene nanoribbon-like structures extending from the CNT framework, together with intact inner CNT cores, confirming selective unzipping of the outer graphitic walls. Atomic-resolution HAADF-STEM imaging directly reveals numerous isolated bright spots corresponding to atomically dispersed Co atoms, together with nanometer-scale Co NPs, clearly demonstrating the coexistence of single-atom and nanoparticle Co sites within the carbon framework (Figure 1d). The corresponding EDS elemental maps further confirm the homogeneous distribution of Co on the surface of the N-doped carbon scaffold (Figure 1e,f).

**Figure 2:**
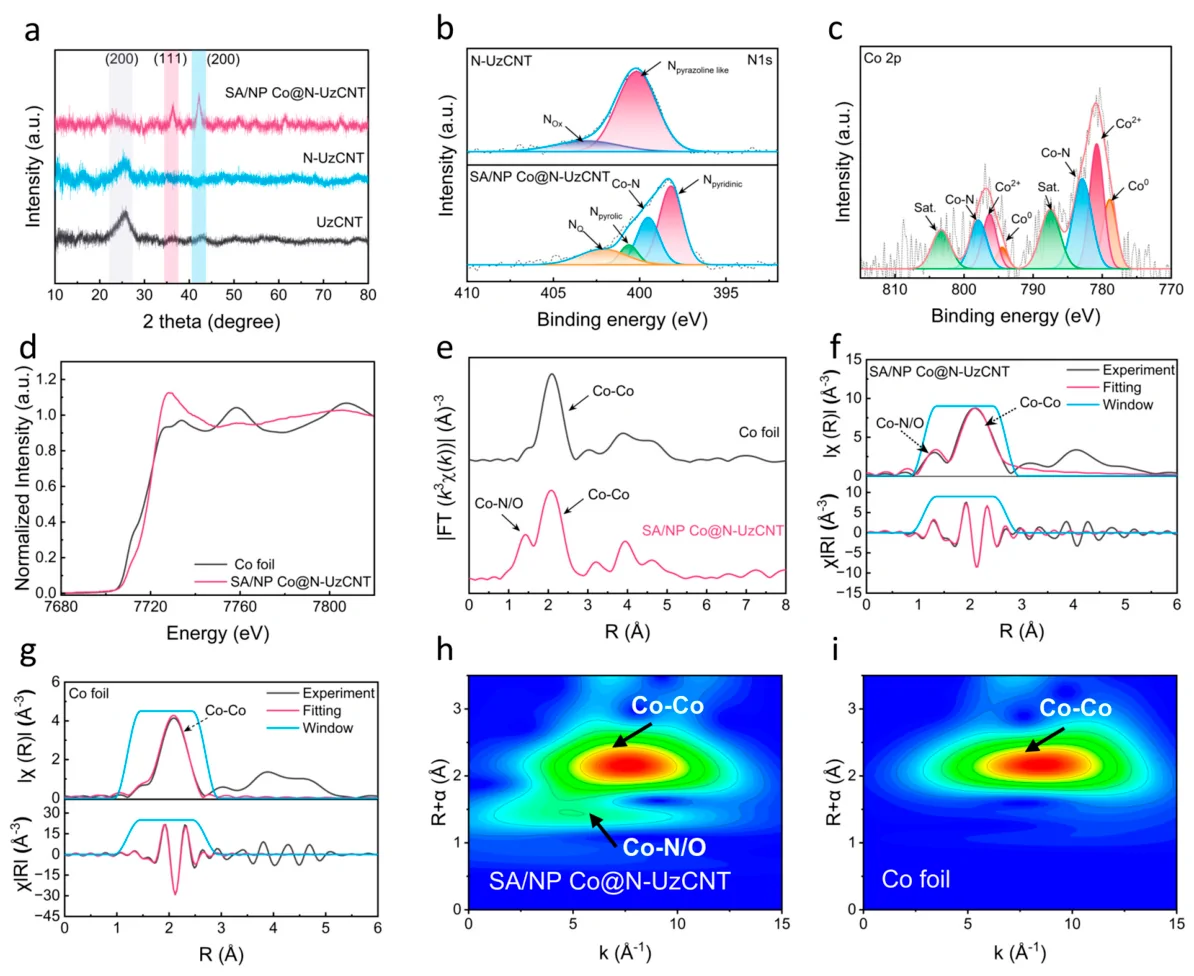
Chemical state and atomic local structure: (**a**) XRD spectra of unzipped CNT, N-doped unzipped CNT and single-atom/nanoparticle cobalt on nitrogen-doped unzipped CNT; (**b**) N 1s XPS spectra of N-UzCNT and SA Co/NP Co@N-UzCNT; (**c**) Co 2p XPS spectra of SA/NP Co@N-UzCNT; (**d**) Co K-edge XANES spectra of SA/NP Co@N-UzCNT and Co foil; (**e**) FT-EXAFS SA/NP Co@N-UzCNT and Co foil. (**f**,**g**) First-shell fitting of the Fourier transform EXAFS spectrum of SA/NP Co@N-UzCNT and Co foil. (**h**,**i**) Wavelet-transform (WT) EXAFS of SA/NP Co@N-UzCNT and Co foil (Red to blue color represent backscattering intensity from high to low).

The structural evolution of UzCNT, N-UzCNT, and SA/NP Co@N-UzCNT was further examined by X-ray diffraction (XRD). As shown in Figure 2a, UzCNT and N-UzCNT exhibit a broad diffraction feature centered at ~25.2°, characteristic of the graphitic carbon framework. In comparison, SA/NP Co@N-UzCNT displays additional peaks at 36.24° and 42.28°, which can be indexed to the (111) and (200) planes of cubic nanoparticle CoO, respectively, indicating the formation of crystalline Co-containing NPs within the carbon framework. Together with the direct observation of nanoscale Co-containing particles by HAADF-STEM, these diffraction features confirm the presence of crystalline CoO nanoparticles in SA/NP Co@N-UzCNT.

The surface chemical states and local bonding environments were investigated by X-ray photoelectron spectroscopy (XPS) (Figure S3 and Table S1). The N 1s spectrum of N-UzCNT can be deconvoluted into components centered at 400.1 and 403.1 eV, assigned to pyrazoline-like and oxidized N species, respectively (Figure 2b). Following Co incorporation and thermal treatment, the N 1s envelope evolves markedly, with contributions from pyridinic-N (399.1 eV), pyrrolic N (400.5 eV), quaternary N (402.1 eV), and an additional Co-N-related component at 399.5 eV. The emergence of the Co-N contribution indicates direct electronic coupling between Co and the N-functionalized carbon framework. In particular, the pronounced pyridinic-N population, together with the Co-N signature, supports the role of edge-localized N functionalities as coordination sites for stabilizing atomically dispersed Co centers. Concurrently, the pronounced decrease in oxygen-containing functional groups in the C 1s spectrum (Figure S4), together with the progressive increase in the I_D_/I_G_ ratio from 0.98 for UzCNT to 1.05 for N-UzCNT and 1.16 for SA/NP Co@N-UzCNT (Figure S5), indicates substantial restoration and reconstruction of graphitic domains during the deoxidative N-doping process [40]. This structural reconstruction is expected to improve the electrical conductivity of the N-UzCNT scaffold while simultaneously generating abundant edge sites favorable for Co coordination.

The chemical states of Co were further evaluated from the Co 2p spectrum (Figure 2c). The Co 2p envelope contains contributions from both metallic and oxidized Co species. Features at 778.9 and 794.4 eV are associated with Co^0^, consistent with the presence of metallic Co domains, whereas components at 780.7 and 796.3 eV are assigned to Co^2+^ species. Additional higher-binding-energy contributions at 782.8 and 797.9 eV, accompanied by characteristic shake-up satellites, reveal a substantial population of electronically perturbed, cationic Co species [41]. The coexistence of metallic and positively charged Co states is consistent with the heterogeneous Co configuration revealed by microscopy, comprising nanoscale Co particles and strongly coordinated Co centers within the N-doped carbon framework. The N 1s and Co 2p spectra provide complementary evidence for strong Co-N interactions that stabilize isolated Co sites and modulate their local electronic structure.

Synchrotron X-ray absorption spectroscopy was employed to directly probe the electronic structure and local coordination environment of the Co species. The Co K-edge X-ray absorption near-edge structure (XANES) spectrum of SA/NP Co@N-UzCNT shows an absorption edge shifted to higher energy relative to Co foil (Figure 2d), revealing an average Co valence state more positive than that of metallic Co. The corresponding Fourier-transformed extended X-ray absorption fine structure (FT-EXAFS) spectrum exhibits a pronounced first-shell contribution at 1.90 Å associated with Co-N/O coordination, accompanied by a Co-Co scattering contribution at 2.49 Å (Figure 2e). In contrast, Co foil is dominated by Co-Co scattering and lacks the pronounced low-R contribution observed for SA/NP Co@N-UzCNT. The simultaneous presence of Co-N/O and Co-Co coordination provides direct structural evidence for chemically distinct Co environments comprising atomically dispersed Co-N sites and nanoscale Co domains, consistent with the HAADF-STEM and XPS analyses. Quantitative EXAFS fitting reproduces both Co-N/O and Co-Co scattering paths, further supporting the coexistence of the two coordination environments (Figure 2f,g and Table S2). This structural heterogeneity is independently visualized by wavelet-transform EXAFS (WT-EXAFS) analysis (Figure 2h,i). In contrast to Co foil, which is dominated by the high-k Co-Co feature, SA/NP Co@N-UzCNT exhibits an intensity maximum at relatively low k values associated with scattering from light N neighbors and a distinct higher-k contribution arising from Co-Co coordination. Taken together, those complementary analyses establish the coexistence of positively charged, N-coordinated Co single-atom sites and Co-containing nanoparticles within the N-UzCNT framework, providing the distinct catalytic motifs underlying the bifunctional electrocatalytic response.

### 3.3. Glucose Sensor Based on Alkali Activation Electrolyte

**Figure 3:**
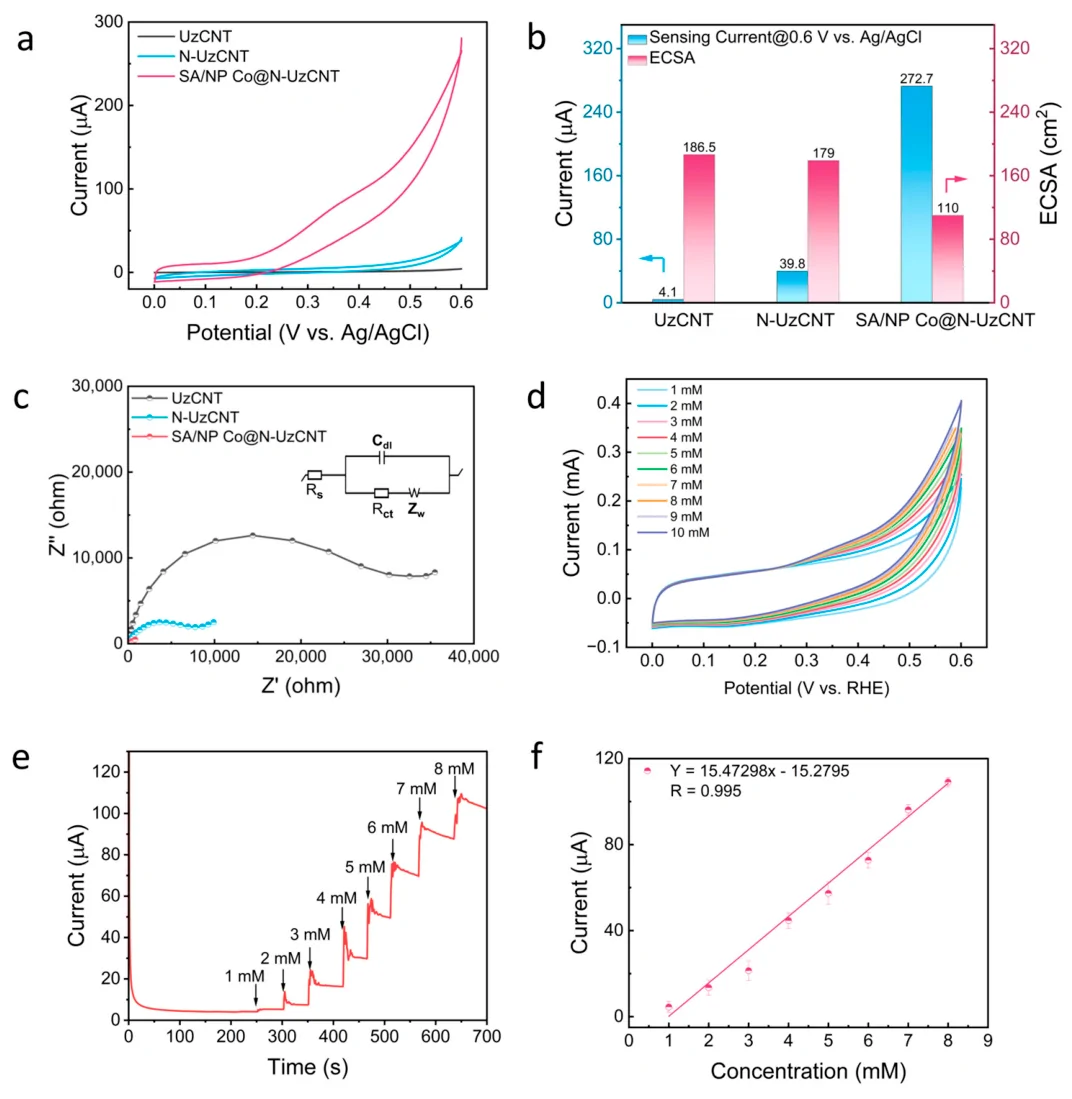
Glucose sensing performance: (**a**) Cyclic voltammograms of UzCNT, N-UzCNT and SA/NP Co@N-UzCNT with 5 mM glucose taken in 0.1 M NaOH at pH 12 at a scan rate of 50 mV s^−1^. (**b**) Sensing responds current and ECSA of UzCNT, N-UzCNT and SA/NP Co@N-UzCNT with 5 mM glucose taken in 0.1 M NaOH at pH 12 at a scan rate of 50 mV s^−1^. (**c**) EIS plot of UzCNT, N-UzCNT and SA/NP Co@N-UzCNT with 5 mM glucose taken in 0.1 M NaOH at pH 12 at a potential of 0.40 V vs. Ag/AgCl. (**d**) CV response at different glucose concentrations from 1 to 10 mM at a scan rate of 50 mV s^−1^. (**e**) Amperometric response of SA/NP Co@N-UzCNT modified electrode to the variation in glucose concentration in 0.1 M NaOH solution at 0.4 V vs. Ag/AgCl. (**f**) Corresponding calibration plot of peak current versus glucose concentration.

The electrocatalytic activity of the prepared electrodes toward glucose oxidation was evaluated by cyclic voltammetry (CV). Figure 3a and Figure S8 compare the CV responses of UzCNT, N-UzCNT, and SA/NP Co@N-UzCNT in 0.1 M NaOH containing 5 mM glucose. Upon glucose addition, SA/NP Co@N-UzCNT exhibits a pronounced increase in anodic current, whereas UzCNT and N-UzCNT show only negligible changes over the investigated potential window. At 0.6 V vs. Ag/AgCl, the oxidation current increases from 4.15 µA for UzCNT and 39.86 µA for N-UzCNT to 272.74 µA for SA/NP Co@N-UzCNT (Figure 3b), demonstrating the substantially enhanced glucose oxidation activity arising from the incorporation of Co species.

To distinguish the contribution of electrochemically accessible surface area from intrinsic catalytic activity, the glucose oxidation current was correlated with the electrochemically active surface area (ECSA) and double-layer capacitance (C_dl_) (Figure S9). The estimated ECSA values of UzCNT, N-UzCNT, and SA/NP Co@N-UzCNT are 186.5, 179, and 110 cm^2^ respectively. Notably, SA/NP Co@N-UzCNT delivers the highest glucose oxidation current despite exhibiting the smallest ECSA among the three electrodes. Rather than arising from a larger accessible surface area, the enhanced response is primarily attributed to the intrinsically higher electrocatalytic activity of the Co-containing active sites.

The strong glucose oxidation activity of SA/NP Co@N-UzCNT is closely associated with the alkaline activation of the Co/CoO species. In an OH^−^-rich electrolyte, surface Co/CoO species undergo electrochemical oxidation to generate high-valence CoOOH, which serves as a redox mediator for glucose oxidation. The overall process can be described by the following reactions:

Co + 2OH^−^ → Co(OH)_2_ + 2e^−^

Co(OH)_2_ + OH^−^ → CoOOH + H_2_O + e^−^ or

CoO + OH^−^ → CoOOH + e^−^

CoOOH + glucose → Co(OH)_2_ + gluconolactone

The electrochemically generated CoOOH species oxidize glucose and are subsequently reduced to Co(OH)_2_, establishing a Co(II)/Co(III) redox cycle under alkaline conditions. This reversible surface redox process activates the Co nanoparticles as catalytic sites for glucose oxidation [42,43].

The critical role of the electrolyte environment was further examined by performing CV measurements in 0.01 M phosphate-buffered saline (PBS). In contrast to the substantial glucose response observed in 0.1 M NaOH, the glucose oxidation current remains negligible in PBS upon glucose addition (Figure S10). The suppression of catalytic activity under neutral conditions is consistent with the limited formation of high-valence CoOOH species in the absence of a sufficiently high OH^−^ concentration. These results establish alkaline electrolyte-induced activation of Co-based surface sites as a prerequisite for efficient glucose oxidation and provide the basis for electrolyte-selective switching of the catalytic function.

Electrochemical impedance spectroscopy (EIS) reveals favorable interfacial charge-transfer characteristics for SA/NP Co@N-UzCNT. As shown in Figure 3c, SA/NP Co@N-UzCNT exhibits the lowest impedance among the investigated electrodes, indicative of reduced charge-transfer resistance. The improved interfacial charge-transfer kinetics promote the electrochemical conversion of Co species and sustain the redox cycling of catalytically active CoOOH during glucose oxidation. The fitted R_ct_ values were ~6.703 Ω for SA/NP Co@N-UzCNT, ~1917 Ω for N-UzCNT, and ~22,674 Ω for UzCNT respectively. The markedly lower R_ct_ value of SA/NP Co@N-UzCNT indicates more favorable interfacial electron-transfer kinetics compared with the control electrodes, supporting the enhanced electrocatalytic performance of SA/NP Co@N-UzCNT. The analytical response of SA/NP Co@N-UzCNT was subsequently evaluated over a range of glucose concentrations. The CV curves show a progressive increase in oxidation current as the glucose concentration increases from 1 to 10 mM (Figure 3d), demonstrating a concentration-dependent electrocatalytic response. Consistently, the amperometric glucose measurements were conducted at an applied potential of 0.4 V vs. Ag/AgCl. This potential was selected based on the electrochemical response observed in the preceding CV measurements, where approximately 0.4 V vs. Ag/AgCl represents the region at which a clear and distinguishable glucose oxidation is exhibited. The amperometric response in Figure 3e exhibits clear stepwise increases in current response upon successive glucose additions. The corresponding calibration plot shows a linear response over the 1–8 mM concentration range, with a sensitivity of 65.43 µA mM^−1^ cm^−2^ (Figure 3f). The analytical performance of SA/NP Co@N-UzCNT is compared with previously reported cobalt-based non-enzymatic glucose sensors in Table S3 [42,43,44,45,46,47,48,49,50]. These results demonstrate sensitive glucose detection by SA/NP Co@N-UzCNT arising from electrolyte-induced activation of Co-based sites and subsequent CoOOH-mediated glucose oxidation.

### 3.4. Uric Acid Sensor Based on Neutral Activation Electrolyte

**Figure 4:**
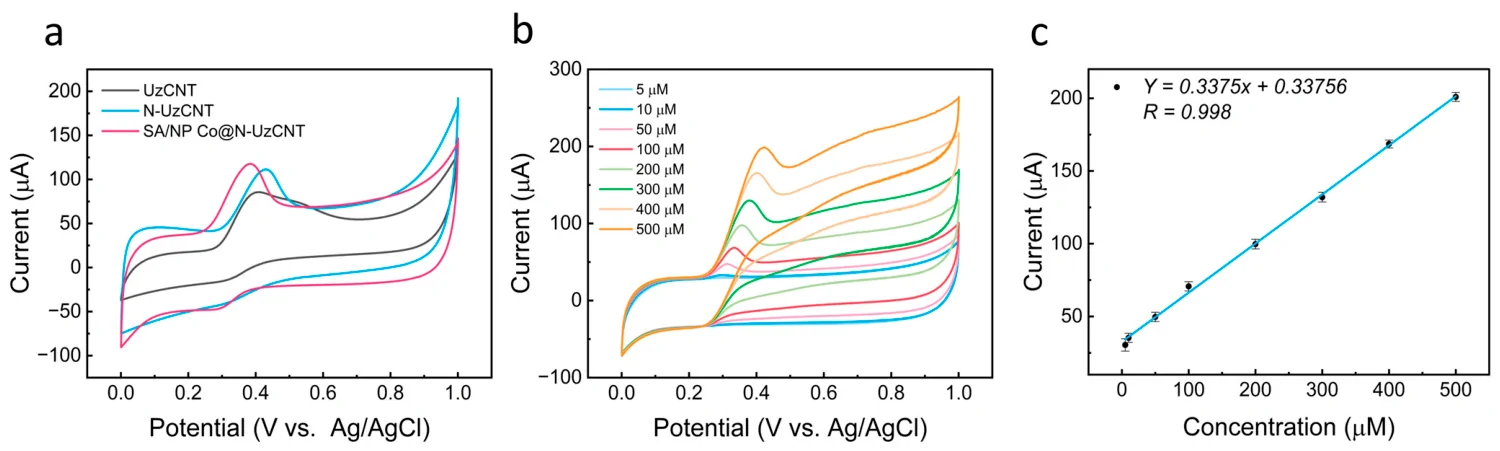
Uric acid sensing: (**a**) CV curves of the UzCNT, N-UzCNT and SA/NP Co@N-UzCNT recorded in a 0.01 M PBS (pH  =  7.5) solution with 400 µM UA; (**b**) CV curves for sample SA/NP Co@N-UzCNT at various uric acid concentrations with a scan rate of 50 mV s^−1^; (**c**) corresponding calibration plot of peak current versus uric acid concentration.

The non-enzymatic sensing performance of SA/NP Co@N-UzCNT toward uric acid (UA) was evaluated under neutral conditions. Figure 4a compares the CV responses of UzCNT, N-UzCNT, and SA/NP Co@N-UzCNT toward UA oxidation in 0.01 M PBS. UzCNT exhibits a weak oxidation response, whereas N-UzCNT shows an enhanced anodic current, suggesting the beneficial effects of edge functionalization and N incorporation on the electrochemical activity of the carbon framework. Notably, SA/NP Co@N-UzCNT displays a substantially intensified and clear oxidation peak at 0.38 V vs. Ag/AgCl, revealing significantly enhanced electrocatalytic activity toward UA oxidation. The enhanced response following Co incorporation is consistent with the participation of atomically dispersed Co-N/O sites in facilitating UA oxidation under neutral conditions. The catalytic response was further examined by comparing the CV profiles of SA/NP Co@N-UzCNT in the absence and presence of UA at a scan rate of 50 mV s^−1^ (Figure S11). Introduction of UA produces a distinct increase in the anodic current, confirming efficient electrochemical oxidation at the SA/NP Co@N-UzCNT interface. Under neutral conditions, where extensive formation of Co oxyhydroxide species is suppressed, the enhanced UA response is attributed predominantly to the atomically dispersed Co-N/O centers embedded within the N-UzCNT framework. The concentration-dependent response of SA/NP Co@N-UzCNT was evaluated by CV in PBS over a potential range of 0–1.0 V vs. Ag/AgCl. The oxidation current gradually increases with increasing UA concentration, exhibiting a linear concentration dependence with a sensitivity of 1420 µA mM^−1^ cm^−2^ (Figure 4b,c and Figure S12). The analytical performance of SA/NP Co@N-UzCNT is compared with previously reported cobalt-based non-enzymatic UA sensors in Table S4 [12,51,52,53,54,55,56,57]. These electronically coupled Co-N/O sites provide favorable adsorption and charge-transfer environments for UA oxidation through a coupled two-electron/two-proton transfer process at the electrode interface (Figure S13), distinguishing their catalytic function from that of the nanoparticle-derived sites activated under alkaline conditions. The contrasting catalytic responses toward UA under neutral conditions and glucose under alkaline conditions highlight the electrolyte-dependent activation of structurally distinct Co sites within a single-electrode architecture.

### 3.5. Practical Application of Glucose and Uric Acid Based Dual Activation Sensor

**Figure 5:**
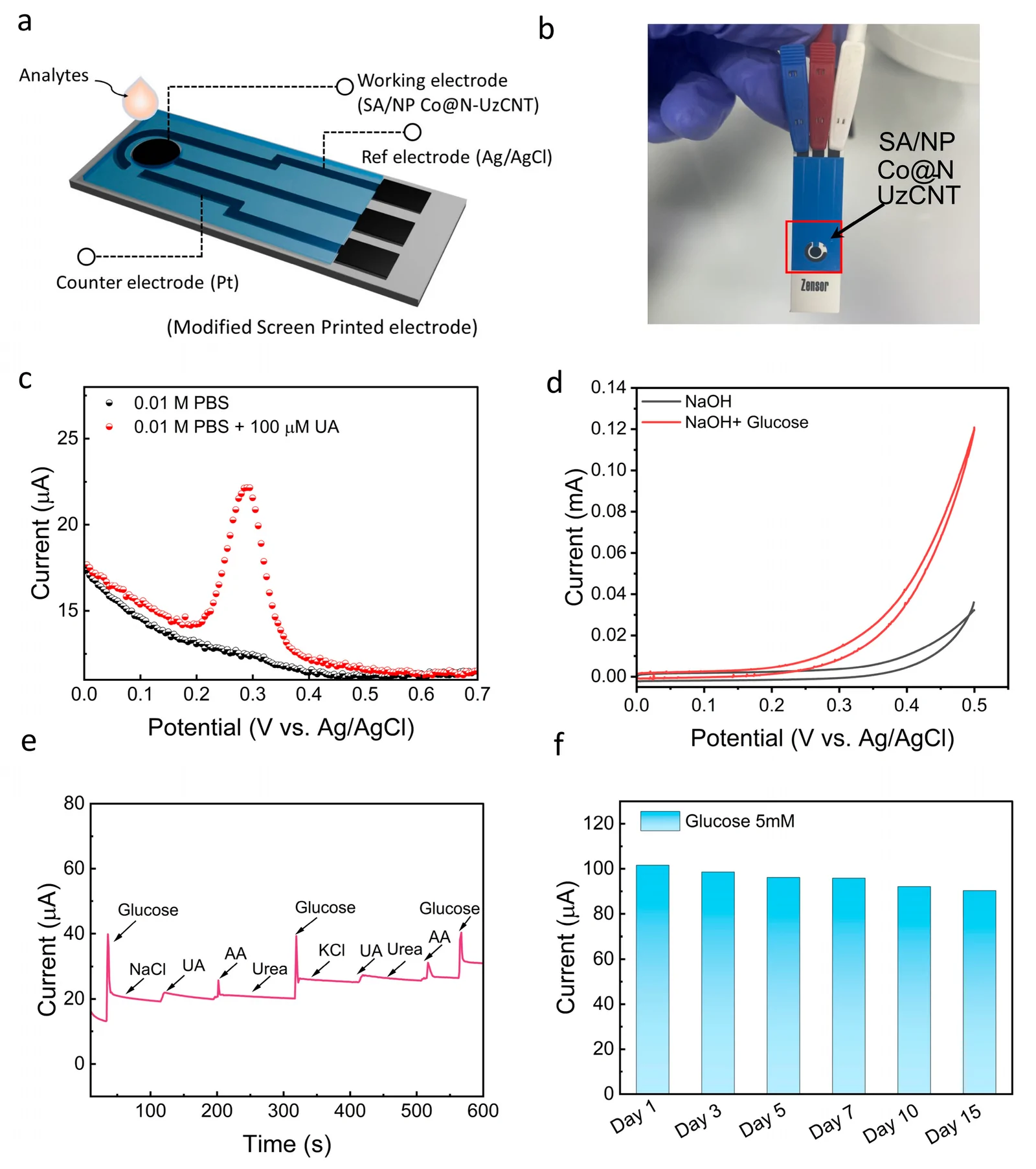
Practical application: (**a**,**b**) Schematic and optical image for screen-printed electrode (SPE) modified SA/NP Co@N-UzCNT. (**c**) DPV for uric acid detection using a screen-printed electrode (SPE) modified SA/NP Co@N-UzCNT in 0.01 M PBS electrolyte. (**d**) CV for glucose detection using a screen-printed electrode (SPE) modified with SA/NP Co@N-UzCNT in 0.1 M NaOH electrolyte. (**e**) Interference assay of SA/NP Co@N-UzCNT with a concentration of 5 mM glucose and interference of 0.5 mM NaCl, 0.5 mM KCl, 0.5 mM urea, 0.1 mM AA and 0.1 mM UA at 0.40 V vs. Ag/AgCl. (**f**) Stability performance of sensor-based SA/NP Co@N-UzCNT.

To demonstrate the practical applicability of the electrolyte-regulated sensing strategy, SA/NP Co@N-UzCNT was integrated onto the working electrode of a screen-printed electrode strip (Figure 5a,b). Remarkably, the same modified electrode could be switched between different target analytes simply by changing the electrolyte environment. In 0.01 M PBS, differential pulse voltammetry (DPV) revealed a distinct anodic peak around 0.28–0.30 V vs. Ag/AgCl following the addition of 100 µM UA, whereas the blank electrolyte exhibited only a weak background response (Figure 5c and Figure S14). The pronounced UA oxidation signal under neutral conditions confirms the electrochemical activity of the SA/NP Co@N-UzCNT electrode toward UA. By contrast, under alkaline conditions, glucose addition produced a substantial increase in anodic current relative to the blank 0.1 M NaOH electrolyte, particularly at higher applied potentials (Figure 5d), consistent with the activation of Co nanoparticle-derived catalytic sites for glucose oxidation. These results demonstrate electrolyte-controlled switching sensing function within a single SA/NP Co@N-UzCNT electrode. Furthermore, repeated switching experiments shown in Figure S15a,b demonstrated the controlled sensing functionality of UA detection in neutral PBS and glucose detection in alkaline electrolyte.

The selectivity of the SA/NP Co@N-UzCNT electrode was further evaluated against representative inorganic ions and electroactive interferents. For glucose detection in alkaline electrolyte, the addition of K^+^, Na^+^, and Cl^−^ produces negligible current changes, while urea, ascorbic acid, and UA generate only minor responses compared with glucose (Figure 5e). Similarly, under UA-sensing conditions, the electrode exhibits minimal responses toward the tested inorganic ions and organic interferents, including glucose; this measurement and reproducibility test are shown in Figure S16a,b. The distinct interference profiles under the two electrolyte environments highlight the ability of the catalyst to preferentially activate different electrochemical pathways for glucose and UA oxidation. This electrolyte-dependent selectivity supports the use of a single catalytic architecture for multi-analyte sensing while minimizing cross-response between the two target analytes. The operational stability of the SA/NP Co@N-UzCNT sensor was assessed by monitoring its electrochemical response toward glucose over 15 days. As shown in Figure 5f and Figure S17, the current decreases gradually from 101 µA on day 1 to 90 µA on day 15, corresponding to retention of approximately 89% of the initial response. The absence of abrupt signal degradation indicates that the electrode maintains stable electrocatalytic activity over the investigated period. The sustained response suggests the structural robustness of the Co single-atom/nanoparticle architecture supported by the N-UzCNT framework, highlighting its potential for durable non-enzymatic sensing applications. This electrolyte-regulated platform establishes a general design principle for programmable multi-analyte sensing by selectively activating structurally distinct catalytic sites within a single-electrode architecture.

## 4. Conclusions

We demonstrated an electrolyte-regulated bifunctional electrocatalyst comprising atomically dispersed Co-N sites and Co/CoO nanoparticles anchored on nitrogen-doped unzipped carbon nanotubes for non-enzymatic multi-analyte sensing. The edge-rich N-UzCNT framework stabilizes structurally distinct Co motifs while preserving an electrically conductive carbon network. Under alkaline conditions, nanoparticle-derived Co sites are electrochemically converted to CoOOH and promote glucose oxidation, whereas atomically dispersed Co-N sites dominate UA oxidation under neutral conditions. This electrolyte-controlled switching enables selective detection of glucose and UA using a single-electrode architecture, with sensitivities of 65.43 µA mM^−1^ cm^−2^ for glucose and 1420 µA mM^−1^ cm^−2^ for UA. For practical validation, the catalysts were integrated into a screen-printed electrode strip, which retained approximately 89% of its initial glucose response after 15 days. Further validation against physiologically relevant interferents, including neurotransmitters, could advance the practical use of this platform toward single-electrode multi-analyte sensing. This work demonstrates that the coexistence of single-atom and nanoparticle sites can be exploited as a functional advantage and establishes electrolyte-dependent active-site switching as a general design principle for programmable non-enzymatic multi-analyte sensing.

## Data Availability

The datasets generated and analyzed during the current study are available from the corresponding author upon reasonable request.

## Supplementary Materials

The following supporting information can be downloaded at https://www.mdpi.com/article/10.3390/s26216777/s1: Figure S1. SEM image of SA/NP Co@N-UzCNTs; Figure S2. TEM image of SA/NP Co@N-UzCNTs; Figure S3. XPS survey spectra for UzCNT, N-UzCNT and SA/NP Co@N-UzCNT respectively; Figure S4. XPS C1s spectra of UzCNT, N-UzCNT and SA/NP Co@N-UzCNT respectively; Figure S5. Raman spectra for UzCNT, N-UzCNT and SA/NP Co@N-UzCNT respectively; Figure S6. Co K-edge FT-EXAFS fitting curves of Co foil in K space; Figure S7. Co K-edge FT-EXAFS fitting curves of SA/NP Co@N-UzCNT in K space; Figure S8. Cyclic voltammogram response with and without glucose of (a) UzCNT, (b) N-UzCNT and (c) SA/NP Co@N-UzCNT in 0.1 M NaOH; Figure S9. CV curves for the constructed sensor toward 50 µM glucose at different scan rates (right) and corresponding linear fitting plots (left) for (a) UzCNT, (b) N-UzCNT and (c) SA/NP Co@N-UzCNT; Figure S10. Cyclic voltammogram of the glucose response of SA/NP Co@N-UzCNT in 0.01 M PBS; Figure S11. Cyclic voltammogram response of SA/NP Co@N-UzCNT with and without UA in 0.01 M PBS; Figure S12. Differential pulse voltammetry for sample SA/NP Co@N-UzCNT at various UA concentrations; Figure S13. The oxidation mechanism of uric acid; Figure S14. Cyclic voltammogram of UA response of SA/NP Co@N-UzCNT in 0.01 M PBS on an SPC strip; Figure S15. (a) Reproducibility test SA/NP Co@N-UzCNTs based glucose sensor on SPE after 10 times repetition. (b) Reproducibility test SA/NP Co@N-UzCNTs based uric acid sensor on SPE after 10 times repetition.; Figure S16. (a) Interference assay of SA/NP Co@N-UzCNT with concentration of 500 μM of UA and interference of 100 μM of glucose, 100 μM of NaCl, 100 μM of KCl, 100 μM of Na_2_SO_4_,100 μM of urea and 50 μM of AA at 0.35 V vs. Ag/AgCl. (b) Interference assay reproducibility test (with concentration of UA 100 μM^*1^, Glucose 100 μM, 500 μM^*2^, 100 μM^*3^); Figure S17 Chronoamperometry stability test for SA/NP Co@N-UzCNT in 0.01 M PBS and 100 mM UA; Table S1. XPS atomic percentages; Table S2. EXAFS fitting; Table S3. Cobalt-based non-enzymatic glucose sensor; Table S4. Cobalt-based non-enzymatic uric acid sensor.
