# Supplementary material for: Cobalt Single-Atom/Nanoparticle Dual Sites on Unzipped Carbon Nanotubes for Electrolyte-Switchable Glucose and Uric Acid Sensing
Source: Sensors (Basel). 2026 Oct 26;26(21):6777. doi: 10.3390/s26216777 (PMC13766190; doi:10.3390/s26216777)
Supplement: Supplementary file 1 [file sensors-26-06777-s001.zip › sensors-4623912-supplementary.pdf]

sensors

MDPI

Article

Cobalt Single-Atom/Nanoparticle Dual Sites on Unzipped Carbon Nanotubes for Electrolyte-Switchable Glucose and Uric Acid Sensing

Alfi Rodiansyah [1], Changhyun Do [2], Sae Yane Paek [3], Jong Min Kim [3,4] and Joonwon Lim [1,4,*]

[1] Department of Information Display, College of Sciences, Kyung Hee University, Seoul, 02447, Republic of Korea; rodiansyahalfi@khu.ac.kr
[2] Laboratoire de Physique de la Matière Condensée, CNRS, École Polytechnique, Institut Polytechnique de Paris, 91128 Palaiseau, France
[3] Extreme Materials Research Center, Korea Institute of Science and Technology, Seoul, 02792, Republic of Korea; jongminkim@kist.re.kr (J.M.K.)
[4] KHU-KIST Department of Converging Science and Technology, Kyung Hee University, Seoul, 02447, Republic of Korea
* Correspondence: joonwon.lim@khu.ac.kr

Supplementary Materials

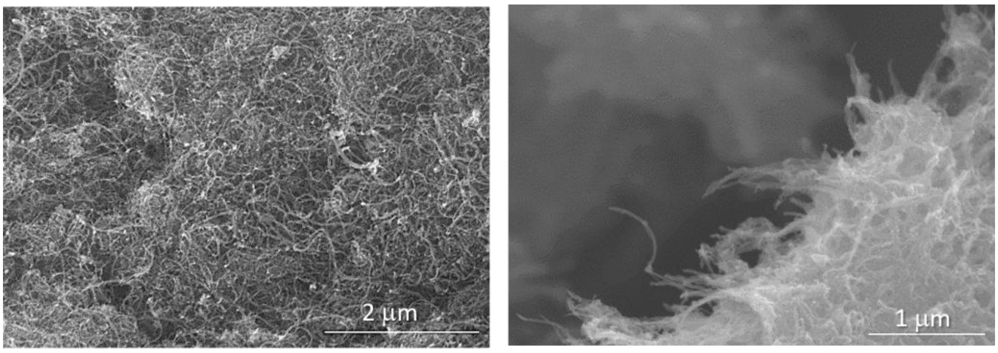

Figure S1. SEM images of SA/NP Co@N-UzCNTs.

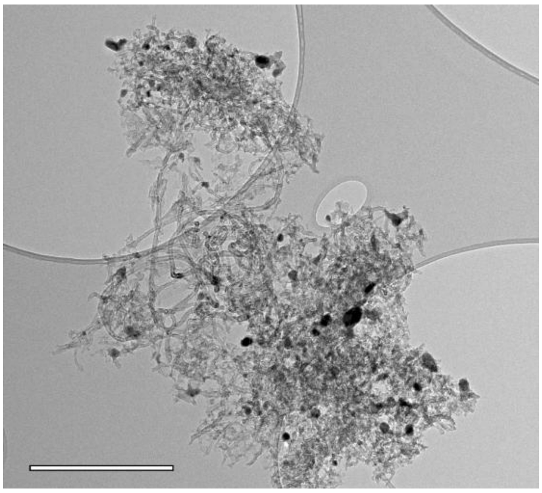

Figure S2. TEM image of SA/NP Co@N-UzCNTs.

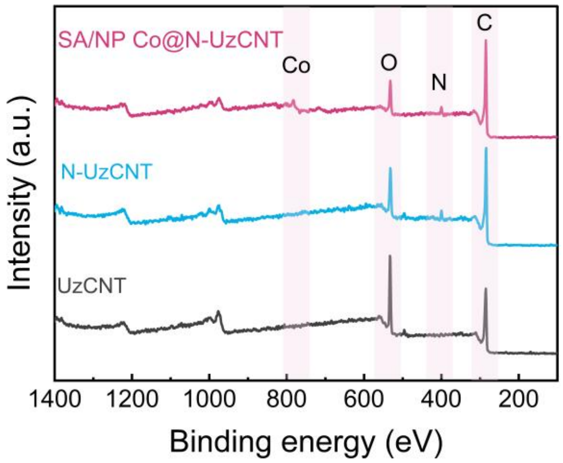

Figure S3. XPS spectra survey for UzCNT, N-UzCNT and SA/NP Co@N-UzCNT respectively.

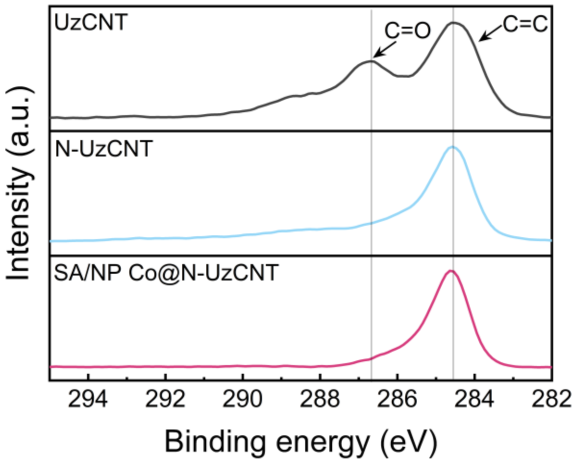

Figure S4. XPS C1s spectra of UzCNT, N-UzCNT and SA/NP Co@N-UzCNT respectively.

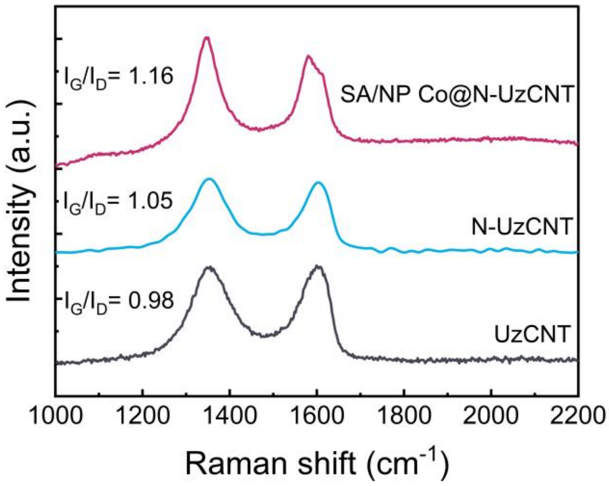

Figure S5. Raman spectra for UzCNT, N-UzCNT and SA/NP Co@N-UzCNT respectively.

https://doi.org/10.3390/s26216777

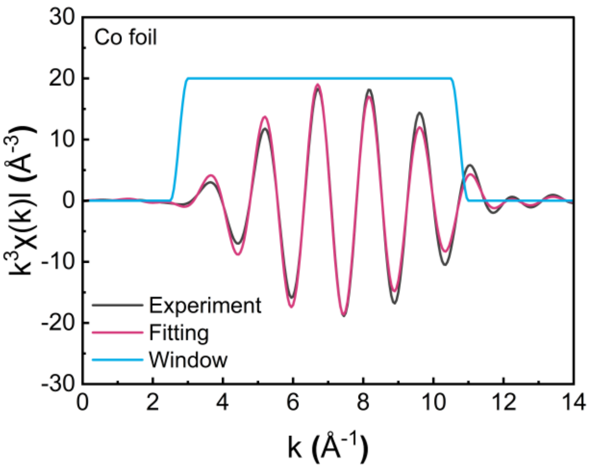

Figure S6. Co K-edge FT-EXAFS fitting curves of Co foil in K space.

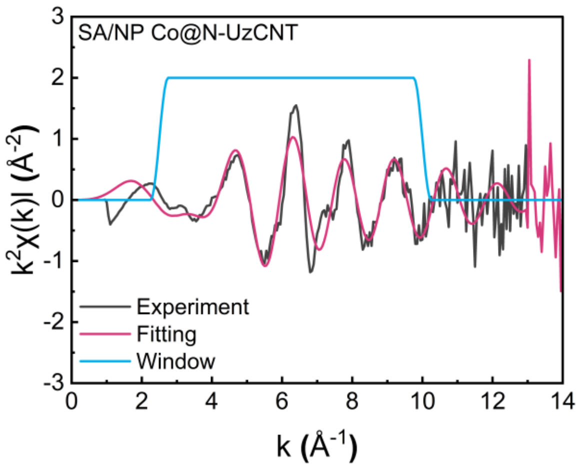

Figure S7. Co K-edge FT-EXAFS fitting curves of SA/NP Co@N-UzCNT in K space.

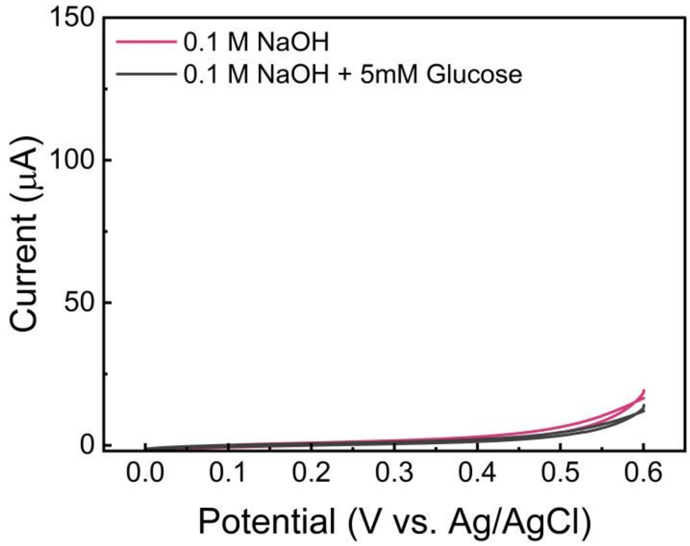

(**a**)

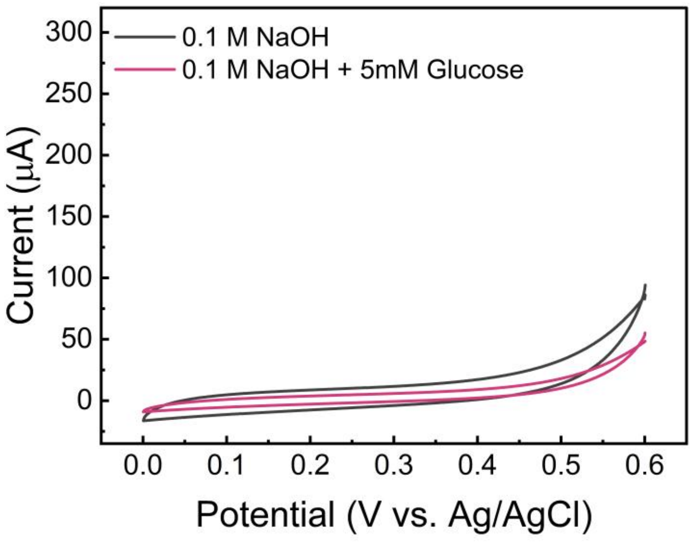

(**b**)

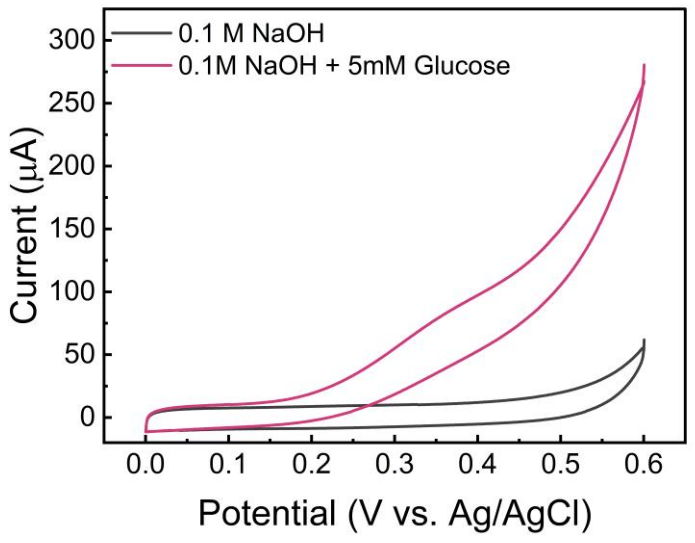

(**c**)

Figure S8. Cyclic voltammogram response with and without glucose of (a) UzCNT, (b) N-UzCNT and (c) SA/NP Co@N-UzCNT in 0.1 M NaOH.

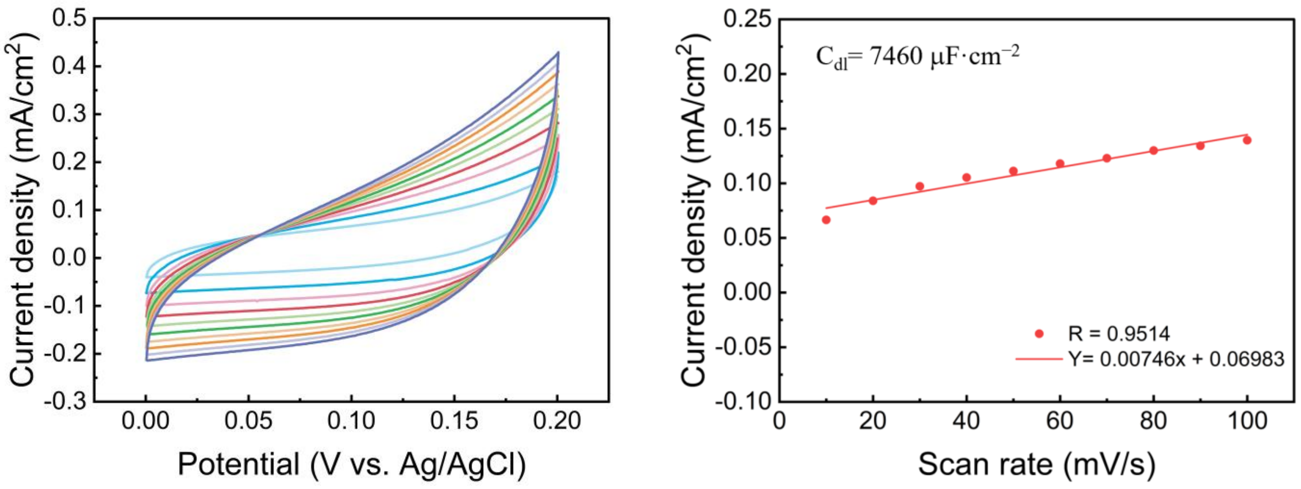

(**a**)

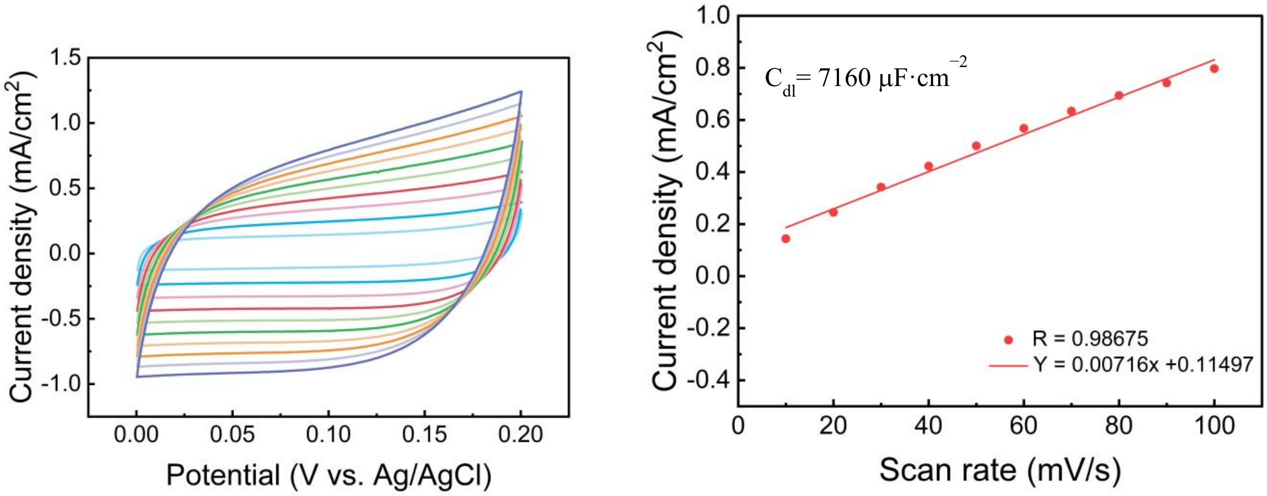

(**b**)

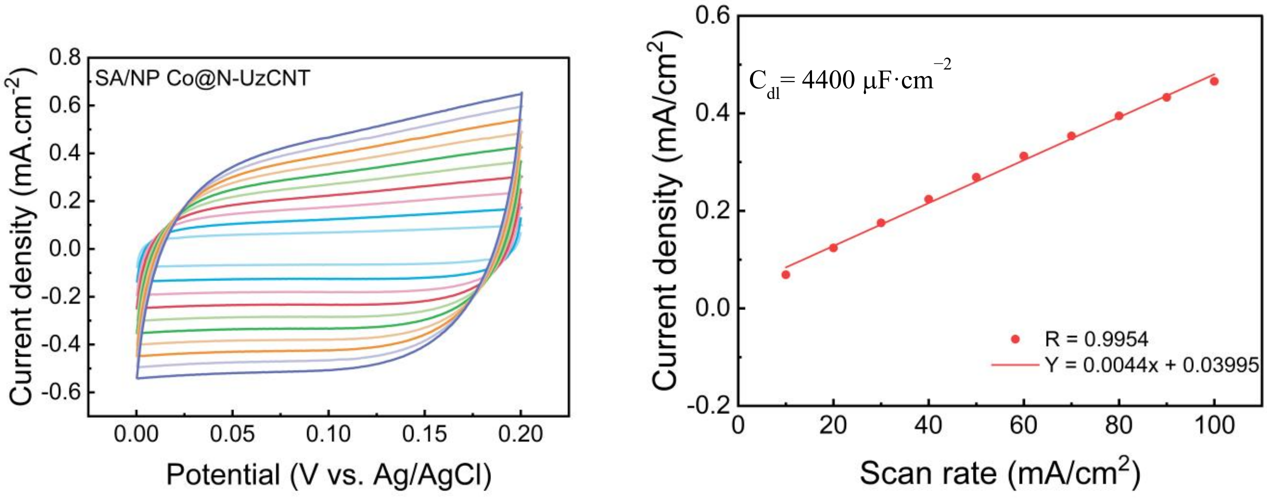

(**c**)

Figure S9. CV curves for the constructed sensor toward 50 μM glucose at different scan rates (right) and corresponding linear fitting plots (left) for (a) UzCNT, (b) N-UzCNT and (c) SA/NP Co@N-UzCNT.

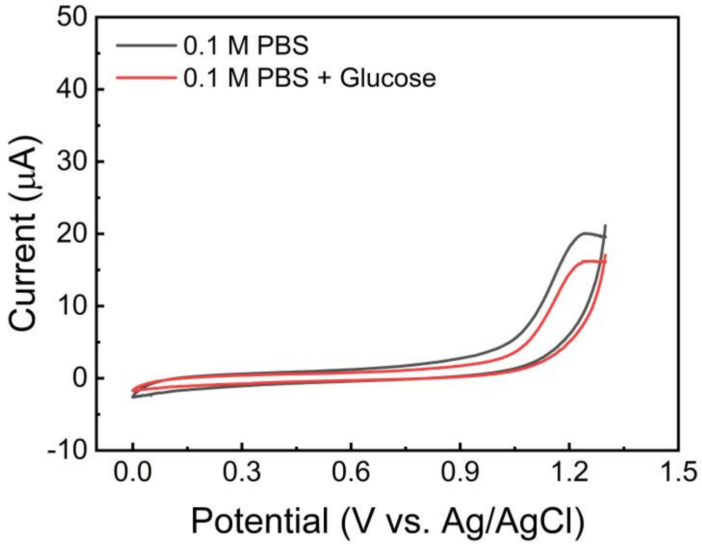

Figure S10. Cyclic voltammogram of the glucose response of SA/NP Co@N-UzCNT in 0.01 M PBS.

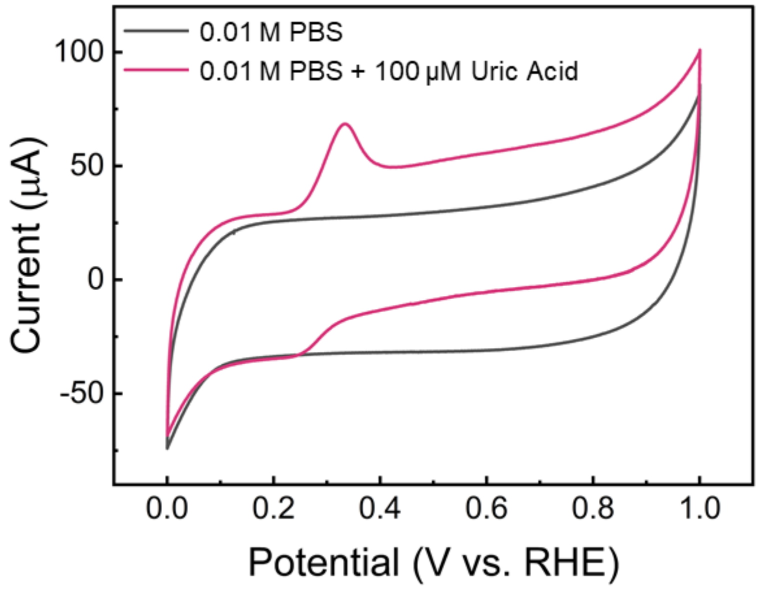

Figure S11. Cyclic voltammogram response of SA/NP Co@N-UzCNT with and without UA in 0.01 M PBS.

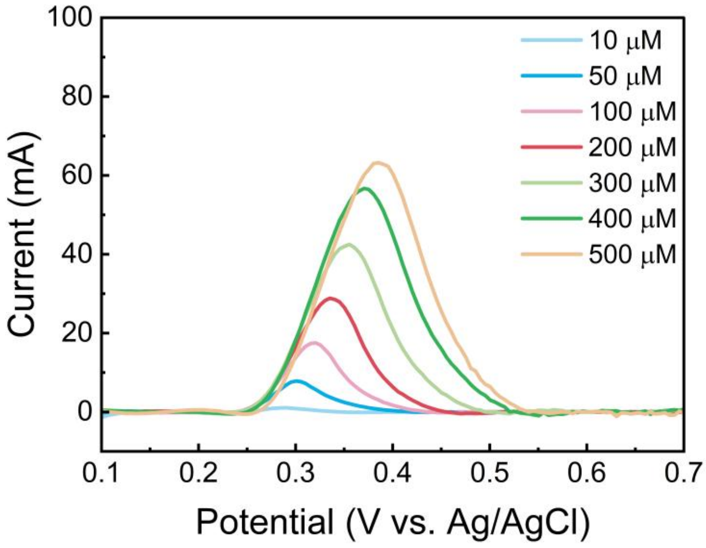

Figure S12. Differential pulse voltammetry for sample SA/NP Co@N-UzCNT at various UA concentrations.

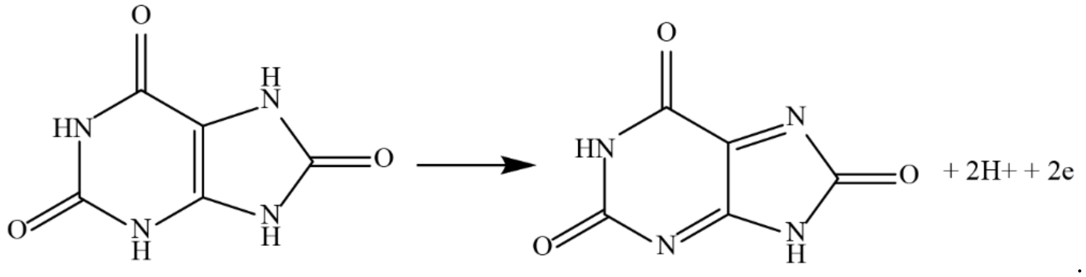

Figure S13. The oxidation mechanism of uric acid.

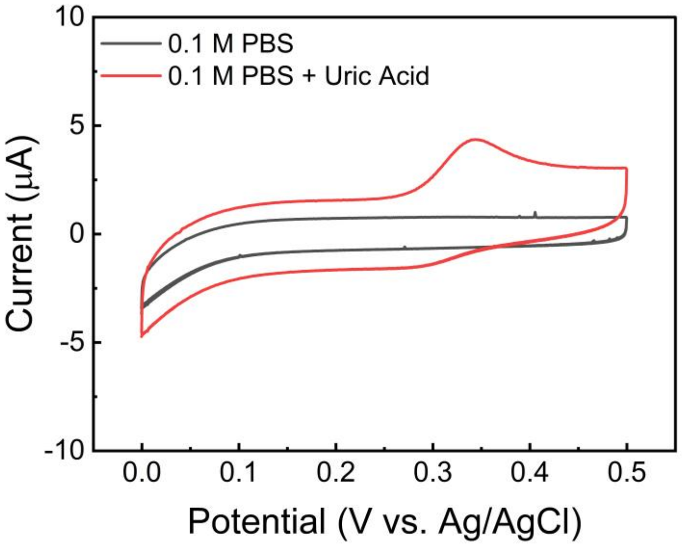

Figure S14. Cyclic voltammogram of UA response of SA/NP Co@N-UzCNT in 0.01 M PBS on an SPC strip.

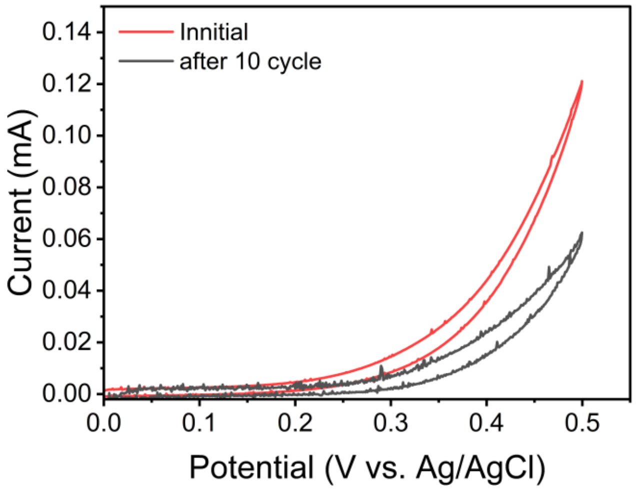

(**a**)

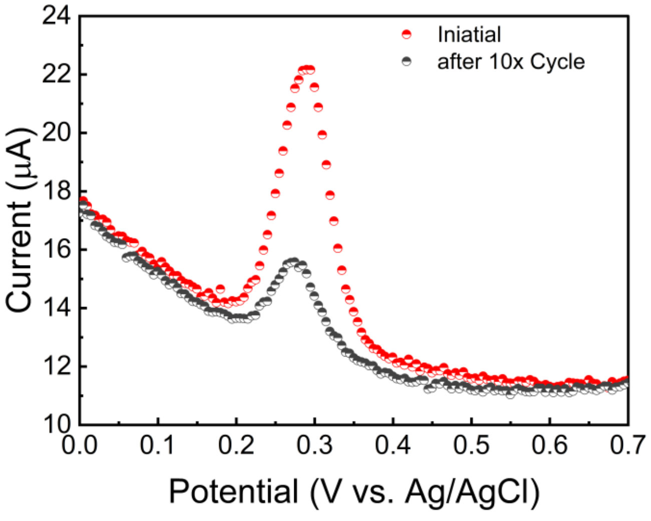

(**b**)

https://doi.org/10.3390/s26216777

Figure S15. (**a**) Reproducibility test SA/NP Co@N-UzCNTs based glucose sensor on SPE after 10 times repetition. (**b**) Reproducibility test SA/NP Co@N-UzCNTs based uric acid sensor on SPE after 10 times repetition.

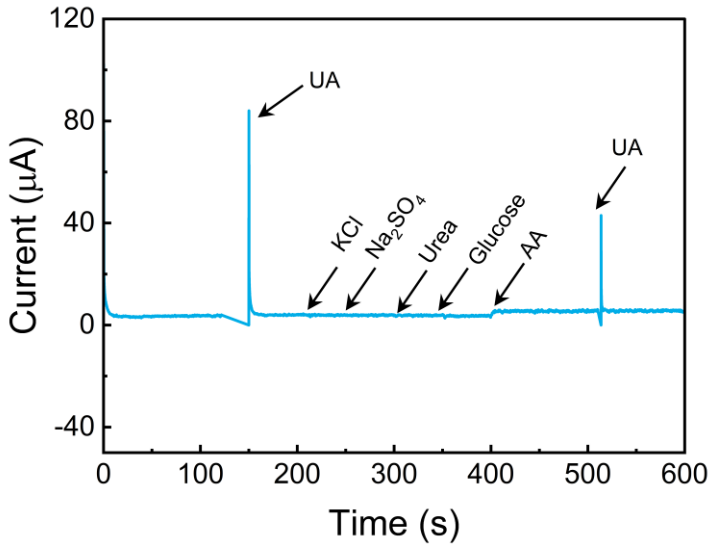

(**a**)

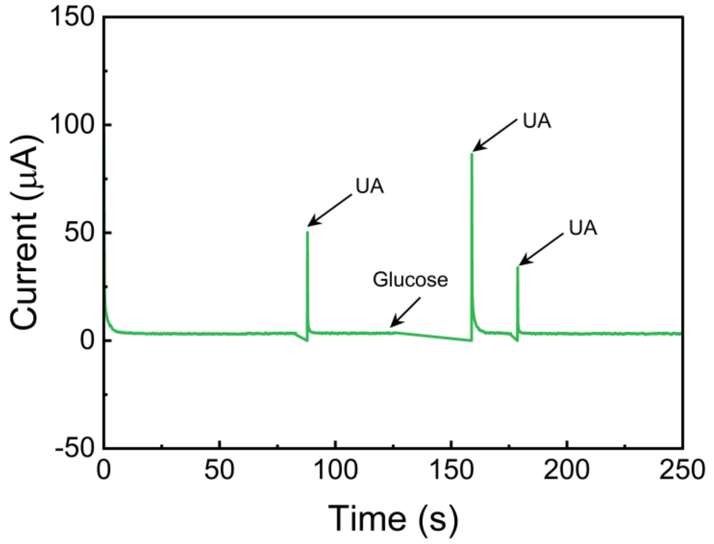

(**b**)

Figure S16. (**a**) Interference assay of SA/NP Co@N-UzCNT with concentration of 500 mM of UA and interference of 100 mM of glucose, 100 mM of NaCl, 100 mM of KCl, 100 mM of Na_2SO_4 ,100 mM of urea and 50 mM of AA at 0.35 V vs. Ag/AgCl. (**b**) Interference assay reproducibility test (with concentration of UA 100 mM[*1], Glucose 100 mM, 500 mM[*2], 100 mM[*3]).

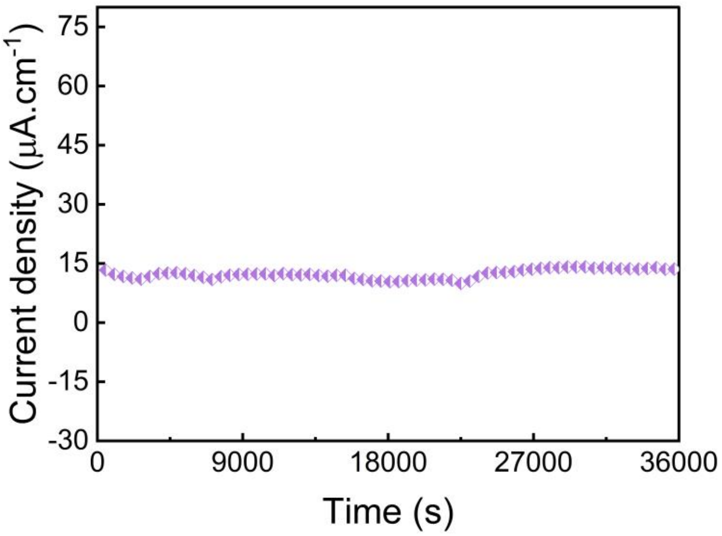

Figure S17. Chronoamperometry stability test for SA/Np Co@N-UzCNT in 0.01 M PBS and 100 mM UA.

Table S1. XPS atomic percentages.

**Samp69.**	**Elements contained (atomic percentage %)**			
	**Carbon**	**Oxygen**	**Nitrogen**	**Cobalt**
UzCNT	69.89	30.14		
N-UzCNT	77.87	17.96	4.17	
SA/NP Co@Uz-NCT	87.64	6.26	5.34	0.76

Table S2. EXAFS fitting

**Sample**	**shell**	**N**	**S_0^2**	**σ^2 (Å^2)**	**DE_0 (eV)**	**R_{eff} (Å)**	**R (Å)**	**R factor**
**Co Foil**	Co-Co	**12**	0.9	0.00877	-4.15	2.49	2.49	0.02
**SA/NP**	Co-N	**2**	0.9	0.00483	-6.11	1.91	1.90	0.01
**Co@N-**	Co-Co	**3**		0.00483	-6.11	2.49	2.49	
**UzCNT**	Co-O	**2.2**	0.9	0.0015	-14.47	1.93	1.93	0.02
	Co-Co	**3.1**	0.9	0.0015	-14.47	2.49	2.49	

N, coordination number; R, distance between absorber and backscatter atoms; σ^2, Debye–Waller factor to account for both thermal and structural disorders; ΔE_0, inner potential correction; R-factor (%) indicates the goodness of the fit. S_0^2 was fixed to 0.9. Fitting range is shown as follows: k = 3-10.5 Å^{-1}, R = 1-3 Å (Co foil), k = 3-11 Å^{-1}, R = 1-2.8 Å for SA/NP Co@N-UzCNT.

Table S3. Cobalt-based non-enzymatic glucose sensor.

**Materials**	**Linear range (mM)**	**Sensitivity (μA mM^{-1} cm^{-2})**	**Technique**	**Electrolyte**	**References**
**Co@NCTs-800**	2-6	72.50	CV	NaOH	43
**Co_3O_4 NDs-GCE**	0.5-5	27.30	CV	NaOH	44
**3D N-Co-CNT@NG**	0.025-10.83	9.05	CV/CA	NaOH	45
**Co/CoO/NC**	0.01-2.75, 2.75-16.65	143.9, 48.0	CV/CA	NaOH	42
**Co_3O_4/MWCNTs/GCE**	0.017–0.554	63.27	CV/CA	NaOH	46
**Co_3O_4/SWCNT/GCE**	1-5	96.92	CV/CA	NaOH	47

**ZIF-67-derived Co_3O_4**	1.5-10.5	50	FET	NaOH	48
**Co-MOF-74 NRs/MC**	0.01-7	98	CV/CA	NaOH	49
**CoCu oxide nanorod arrays/CF**	0.001-1.07	11.90	CV/CA	NaOH	50
**SA/NP Co@N-UzCNT**	**1-8**	**65.18**	**CV/CA**		**Our work**

Table S4. Cobalt-based non-enzymatic uric acid sensor

Electrode material	Linear range (μM)	Sensitivity (μA mM^{-1} cm^{-2})	Technique	Electro-lyte	Ref.
**Co_3O_4/GO**	0.04-1217.2		CV/DPV	PBS	51
**A-Co-NG single-atom catalyst**	0.4-1055 / 1055-41,950	297.2 / 21.2	CA/CV	NaOH	12
**Silky Co_3O_4/GCE**	500-3500		CV	PBS	52
**Co_3O_4 nanoberries/GCE**	5-3000	206	CV	PBS	53
**Porous Co_3O_4 nanosheets/GCE**	0-800	3566.5	DPV	PBS	54
**Hexagonal Co_3O_4 nanosheets/SPCE**	5-200	1560	CV	PBS	55
**Co_3O_4 puffy balls/GCE**	0-1500	2158/307	DPV/CV	PBS	56

https://doi.org/10.3390/s26216777

**Co oxide/porous carbon@MWCNT**	1-40	2140	DPV	PBS	57
**SA/NP Co@N-Uz-CNT**	**5-500**	**337.5**	**CV/DPV**	PBS	**This work**
